# The gut-brain axis and anxiety: investigating associations with diet, vigorous activity, and prior adversity

**DOI:** 10.3389/fpsyg.2026.1815958

**Published:** 2026-07-01

**Authors:** Jenna Louise Pennella, Paul T. Fuglestad

**Affiliations:** Department of Psychological and Brain Sciences, University of North Florida, Jacksonville, FL, United States

**Keywords:** anxiety, diet, GI symptoms, gut-brain axis, prior adversity, vigorous activity

## Abstract

**Introduction:**

The current study investigated associations between different stressors (e.g., poor diet, vigorous activity, and prior adversity), GI symptoms, and anxiety.

**Methods:**

We collected survey data from a sample of university students and community participants (*N* = 209).

**Results:**

Key findings included: (a) vigorous activity was a significant moderator of the relationship between stress and GI symptoms; (b) GI symptoms significantly mediated the relationships between stress and anxiety and prior adversity and anxiety; (c) stress and GI symptoms were significant serial mediators of the relationships between diet quality and anxiety and prior adversity and anxiety.

**Discussion:**

Our findings suggest that factors such as adversity, diet, and physical activity levels should be considered when examining gut-brain relations specific to stress, anxiety, and GI symptoms. Future research should investigate the directionality of these relationships more clearly through longitudinal and experimental study designs.

## Introduction

The gut has been found to have a complex bidirectional relationship with the brain through multiple pathways (Asadi et al., 2022; Góralczyk-Bińkowska et al., 2022). Recent work has investigated this bidirectional relationship to further determine what aspects of each system affect one another, incorporating variables such as diet, exercise, mental health, and stress. While the current literature shows a general understanding of connections between the gut and the brain when taking these variables into consideration, there are additional complexities between these variables that need to be understood with respect to implications for better gut-brain relationships. Specifically, it is important to understand the ways in which variables such as stressors, lifestyle behaviors, and the gut-brain axis interact with each other to know how to move forward in treating related negative symptoms.

### Stress and the gut-brain axis

Stress processes play a large role in the bidirectional nature of the gut-brain connection. Stress hormones are regulated in the body through the hypothalamic–pituitary–adrenal axis (HPA axis) in a negative feedback loop (Hill et al., 2008; Leistner and Menke, 2020). When presented with a stressor, the sympathetic system triggers the HPA axis to release the stress hormone cortisol, and the parasympathetic system, activated by the vagus nerve, reduces the release of cortisol. While the HPA axis is meant to regulate the physiological responses of these stressors, excessive levels of stress may lead to dysregulation in this bodily function (Bonaz et al., 2018; Herman et al., 2016).

Although dysregulation in the HPA axis can come from experiencing stress chronically, it may also be triggered by inflammation in the gut, further explaining a bidirectional relationship between the gut and the brain. Some external signs of gut inflammation include diarrhea, stomach pain, loss of appetite, and fatigue (Mayo Foundation for Medical Education and Research, 2024). Inflammation may occur through excessive and chronic stress, triggering the overgrowth of inflammatory bacteria within the gut (De Punder and Pruimboom, 2015). The vagus nerve connects the gut and the central nervous system and is responsible for triggering the parasympathetic response in the body. Inflammation may lead to dysregulation of the vagus nerve, making the parasympathetic response inaccessible, which guts the negative-feedback loop of the HPA axis (Bonaz et al., 2018).

Thus, the current literature shows that gut issues and external stressors are two potential mechanisms for dysregulated cortisol release, both with the ability to make the other worse, mimicking a positive feedback loop (Bonaz et al., 2018; De Punder and Pruimboom, 2015). Therefore, it is important to identify potential causes of inflammation and chronic stressors. Considering prior literature, some preventative factors may include a balanced, fiber-rich, low-processed diet (Bailey and Holscher, 2018; Madison and Kiecolt-Glaser, 2019; Perler et al., 2023), balanced levels of physical activity (Anderson and Wideman, 2017; Gottschall et al., 2020), and stress-relieving practices such as mindfulness (Allexandre et al., 2016; Gardi et al., 2022). Examining the relations between dietary factors, physical activity, and mental health may deepen understanding of the gut-brain axis.

### Dietary factors and the gut-brain axis

Previous studies have found that the Western diet and lifestyle, consisting of high-fat consumption, processed foods, and high animal protein is harmful for the gut microbiome and stress (Madison and Kiecolt-Glaser, 2019). Specifically, the Western diet may contribute to systemic inflammation (Malesza et al., 2021), and maintaining a poor diet contributes to gut dysbiosis and harmful changes in microbiota (Campaniello et al., 2022; de Wit et al., 2012; Tomasello et al., 2016). However, a Mediterranean diet may be beneficial for gut-health and inflammation (Leeuwendaal et al., 2022; Perler et al., 2023). A Mediterranean diet consists of a well-balanced, diverse diet with healthy oils (e.g., extra-virgin olive oil, wild-caught salmon, avocado), vegetables high in fiber and proteins, and minimal red meats and processed foods (Kiani et al., 2022). This diet supports anti-inflammatory effects (Perler et al., 2023), which may be beneficial in one’s ability to regulate stress.

Two influential factors in the Mediterranean diet that improve gut-health are high fiber intake and low consumption of saturated fat relative to unsaturated fats (Bailey and Holscher, 2018). Specifically, foods such as extra-virgin olive oil, seeds, nuts, non-starchy vegetables and fruits, fish, and legumes, which are rich in fiber and unsaturated fats, are main components of the Mediterranean diet (Kiani et al., 2022). In contrast, Western diets consisting of high levels of saturated fats and low fiber are associated with gut dysbiosis and lower microbial diversity (de Wit et al., 2012). Because of the interconnectedness of the gut and brain, gut dysbiosis may be harmful in terms of inflammation and dysregulated cortisol release.

High dietary fiber, which mainly comes from low-starch vegetables and fruits, has been shown to enrich beneficial gut microbes and decrease gut pathogens (Chen et al., 2023). Consumption of these foods is beneficial because fiber cannot be digested, so it is fermented within the large intestine to produce these bacteria (Holscher, 2017). Thus, fiber helps in maintaining a healthy gut microbiome, supporting anti-inflammation within the gut. Given the relationship between gut inflammation, stress, and regulation of the HPA axis, it would be expected that a poor diet, low in fiber and high in saturated fats, has the potential to dysregulate stress responses through inflammation. However, few studies have investigated the impact of poor diet on stress regulation through indications of gut-health.

### Vigorous activity and the gut-brain axis

Moderate levels of exercise, about 30 to 60 min, 3 to 5 days per week (Garber et al., 2011), have been shown to reduce cortisol release over time. That is, it creates a spike of cortisol during physical activity, then allows the body to better regulate the stress hormone (Anderson and Wideman, 2017). However, exercise that is strenuous and consistent may cause HPA axis dysregulation. One study that measured salivary levels of cortisol suggested that individuals who regularly exercise should not surpass 4–9% of their training being above 90% maximum heart rate to avoid symptoms of overreaching (Gottschall et al., 2020). The relations with high-intensity exercise and higher cortisol levels (Hill et al., 2008; Vanbruggen et al., 2011) suggest that athletes who take part regularly in high-intensity cardiovascular forms of physical activity may be at risk for dysregulation of stress.

Contrary to consistent moderate exercise, previous research has also shown levels of vigorous activity to be associated with higher levels of gut permeability, allowing more pathogens to enter the bloodstream (Clauss et al., 2021). These pathogens may increase the levels of inflammation within the gut, potentially leading to dysregulation in the HPA axis (Bertollo et al., 2025; Fukui, 2016). Multiple studies have also shown a relationship between consistent vigorous activity and gastrointestinal (GI) distress, a sign of inflammation in the gut (Clark and Mach, 2016; Wilson, 2018). This research poses interesting questions about the interplay of gut-health and physical activity with stress levels, given that both gut systems and physical activity can trigger the release of cortisol. Specifically, is vigorous activity associated with GI distress, and if so, what effects does that have on one’s mental health?

### Gut-health and anxiety

There is a well-established connection between stress and anxiety, and as discussed, a well-established connection between stress and differing aspects of gut-health. Anxiety, which is often comorbid with depression, is associated with higher levels of stress (Mihić et al., 2023), which may potentially be explained by external or bodily stressors. Specifically, greater prior adversity is associated with higher anxiety levels (Goodman et al., 2022; Kim et al., 2023), and gut inflammation and anxious behaviors have been linked in non-human subjects (Zou et al., 2023). As discussed, bodily inflammation may be triggered by external stressors, but also poor diet or strenuous physical activity. Because of the associations between anxiety, stress, and gut-health, one might expect to see the relationship between stress and anxiety be altered through differing levels of gut-health, which may be explained through diet or physical activity. Alternatively, it may be expected that differing perceived stress levels alter the relationship between gut-health and anxiety.

Given the bidirectional nature of the relationship between the gut and the brain, it is important to note that interventions could target external stressors to improve digestive health. As discussed above, external stressors may impact the gut through inflammation of the vagus nerve and dysregulation in the HPA axis. However, symptoms of depression and anxiety may also worsen GI symptoms in similar ways. Research has found high inflammation in depressed individuals (Bremner et al., 2020), and one study showed that depressed individuals had an overactive HPA axis compared to non-depressed participants (Peirce and Alviña, 2019). Additionally, anxiety was found to be positively correlated with GI distress in runners, and, interestingly, perceived exertion was also positively correlated with GI distress (Wilson, 2018). These prior findings suggest there are connections between gut-health, mental health, and stress. The current study seeks to piece together these pathways, investigating specific antecedents related to anxiety through the autonomic nervous system.

### Current study

The background literature shows a bidirectional relationship between stress and the gut, in that high levels of external stress may lead to gut dysbiosis and inflammation, and gut inflammation and dysbiosis may lead to high levels of bodily stress through dysregulation of the vagus nerve and HPA axis (Bonaz et al., 2018; De Punder and Pruimboom, 2015). However, there is a lack of literature that examines the relationships of diet and physical activity level with mental health through GI symptoms and stress. Previous literature suggests that the relationship between diet/physical activity and mental health exists (Wilson, 2018; Zhao et al., 2020), as well as pointing out their relationships with gut-health and stress (Bonaz et al., 2018; De Punder and Pruimboom, 2015). However, sparse literature bridges the gaps between these two areas of study.

The current study aimed to examine the relationships between stress, GI symptoms, and anxiety to better understand how mental health is related to the stressors discussed. Based on prior literature, we hypothesized that (1) vigorous activity and diet would moderate the relationship between stress and anxiety and stress and GI symptoms (i.e., stronger associations when vigorous activity is high and diet is poor); (2) GI symptoms would mediate the associations between variables of interest (i.e., diet, stress, physical activity, prior adversity) and anxiety; and (3) perceived stress and GI symptoms would serially mediate the associations of diet, physical activity, and prior adversity with anxiety.

## Method

### Participants

Participants were recruited via the Sona System (*n* = 177) and a community participant pool (*n* = 32) in the southeastern United States (see Table 1). Individuals recruited through the Sona System were students from a university who received class credit for participation. Community pool participants were community members who opted to be contacted for potential research opportunities. They received $5 for their participation. All participants completed informed consent, and all measures and procedures were approved by the university Institutional Review Board.

**Table 1:** Demographics of the current sample (*N* = 209).

	*M* (SD)	*n* (%)
Age	24.03 (10.67)	
Biological sex		
Male		33 (15.8)
Female		176 (84.2)
Gender		
Male		29 (13.9)
Female		173 (82.8)
Non-Binary		6 (2.9)
Race/Ethnicity		
American Indian/Alaska Native		2(0.9)
Asian		12 (5.7)
Black/African American		32 (15.1)
Hispanic/Latinx		14 (6.6)
White		123 (58.0)
Mixed Race/Ethnicity		26 (12.3)
BMI	26.09 (6.30)	

### Materials

#### Diet

Diet was assessed with the Short Form Food Frequency Questionnaire (SFFFQ) (Cleghorn et al., 2016), developed in the United Kingdom. This questionnaire is a short form developed from the Food Frequency Questionnaire (FFQ) (Cade et al., 2004), which is a widely used questionnaire to assess diet quality. Cleghorn et al. (2016) found the SFFFQ and FFQ to not significantly differ from each other in terms of dietary quality score (DQS). Because the scale was used with an American sample, some modifications were made to ensure participants understood questions (e.g., ‘chicken twizzlers’ → ‘chicken nuggets’; ‘crisps’ → ‘chips’; ‘tinned’ → ‘canned’). The measure includes questions regarding typical frequency of food and beverage consumption over a week during the past month. Scoring for dietary quality was split into five different categories representing intake of fruit, vegetables, oily fish, fat, and sugar. Each of those categories was scored by either a 1, 2, or 3, representing low, moderate, or high-quality intake for each category. Additionally, an overall diet quality score, which was comprised of the categories mentioned, was calculated.

#### Physical activity

Physical activity was measured using the International Physical Activity Questionnaire–Short Form (IPAQ-SF), which has been shown to have acceptable internal reliability and validity (Craig et al., 2003). This questionnaire asks questions about physical activity in the last 7 days to gauge levels of activity in a typical week. The questionnaire asks frequency questions regarding the time spent doing sedentary, moderate, or vigorous activity, asking days per week, hours, and minutes. Scores are computed by getting individual scores for walking, moderate, and vigorous categories. For the purpose of this study, we utilized the vigorous activity measure, with higher scores indicating higher frequency of vigorous activity in 1 week. Along with the scale’s reliability and validity, the vigorous activity subscale was chosen due to the feasibility of a continuous score created to measure the amount of vigorous activity over a week. As discussed above, vigorous activity specifically has been shown to have different effects on the HPA-axis, as compared to moderate or sedentary activity. Therefore, a composite score for overall physical activity would not align with the current study aims. It is important to note that this scale was validated as a composite measure, so the use of the vigorous activity subscale should be further validated. However, this subscale is built to effectively capture the amount of vigorous activity an individual performs in 1 week.

#### GI symptoms

GI symptoms were measured using the Gastrointestinal Symptom Rating Scale (GSRS) (Kulich et al., 2008). The GSRS has shown acceptable test–retest reliability and internal consistency reliability. This scale includes 15 items that ask about the level of discomfort for different GI symptoms over the past week (i.e., heartburn, acid reflux, gas, constipation). Level of discomfort is measured on a 7-level scale from *no discomfort at all* to *very severe discomfort*. Responses from each item were summed, and higher scores were representative of higher GI symptoms.

#### Anxiety

The Beck Anxiety Inventory (BAI) was used to measure anxiety in the past month (Beck et al., 1988). The scale includes 21 items that ask questions indicating how much the individual has been bothered by listed symptoms (e.g., heart pounding/racing, hands trembling, fear of losing control). Each item was scored from 0 (*not at all*) to 3 (*severely*), and items were summed to form a scale score. Scores from 0–21 are considered low anxiety, 22–35 moderate anxiety, and 36 + potentially concerning levels of anxiety. The scale has demonstrated good internal reliability and validity, correlating moderately with the Hamilton Anxiety Rating Scale and Hamilton Depression Rating Scale (*r* = 0.51, *r* = 0.25) (Beck et al., 1988).

#### Stress

The 10-item Perceived Stress Scale (PSS) was used to measure perceptions of stress (Cohen and Williamson, 1988). This scale assesses thoughts and feelings during the last month that pertain to feelings of stress. Questions are rated on a scale from 0 (*never*) to 4 (*very often*). The items were summed to form a scale score. High PSS scores are correlated with failure to quit smoking, vulnerability to a prior stressful event eliciting depressive symptoms, and more colds (Cohen and Williamson, 1988).

#### Prior adversity

The Adverse and Traumatic Experiences Scale (Dale et al., 2020) was used to measure prior adversity. This scale was created using a combination of items from other measures regarding traumatic history and prior life events (i.e., ACES, Felitti et al., 1998; Trauma History Questionnaire, Hooper et al., 2011; Brief Trauma Questionnaire, Schnurr et al., 1999; Life Events Checklist for DSM-5, Weathers et al., 2013). The scale includes 30 items and is split into seven different categories: childhood adverse experiences, childhood maltreatment, intimate partner maltreatment, other person maltreatment, life-threatening situations, sudden losses, and personal health situations. The response scale ranges from 0 (*did not occur*) to 4 (*big impact on my life*). Scores for each item were summed together, and higher scores were suggestive of higher prior adversity.

### Analysis plan

#### Power analysis

Power analyses were performed to set a target sample size. GPower was used to establish the sample size needed to detect an *R*^2^ increase of 0.03 due to an interaction (e.g., diet quality x stress → anxiety). To achieve 80% power, we would need 256 participants. The pwr2ppl package in R was used to estimate the sample size needed to investigate mediation effects. Based on prior literature (e.g., Goodman et al., 2022; Hilimire et al., 2015; Lee and Kim, 2019; Wilson, 2018), we expected associations between predictors, mediators, and outcomes (e.g., diet → gut-health → anxiety; adversity → stress → anxiety) in the small to medium range (*r*’s 0.2 to 0.6). To achieve 80% power to detect simple mediation with associations of *r = 0*.3, we would need 150 participants. To achieve 80% power to detect simple mediation with associations of *r = 0*.2, we would need 310 participants.

*Hypothesis 1*: We used multiple linear regression through SPSS Process Model 1 to examine physical activity and diet as separate moderators of the association between stress and anxiety. Additionally, we used the same procedure to examine physical activity and diet as separate moderators of the association between stress and GI symptoms.

*Hypothesis 2*: We analyzed associations between various predictors (e.g., diet, stress, physical activity, prior adversity) and anxiety through GI symptoms using SPSS Process Model 4. We examined the direct and indirect relationships to determine the effect size of GI symptoms as a mediator for the various predictors and anxiety.

*Hypothesis 3*: We examined perceived stress and GI symptoms as serial mediators of the associations of diet, physical activity, and prior adversity with anxiety. Specifically, we used SPSS Process Model 6 to separately examine serial mediations with stress as the initial mediator, followed by GI symptoms, with either diet, physical activity, or prior adversity as the antecedent variable, and anxiety as the outcome variable.

## Results

### Moderating effects of physical activity and diet

Diet did not moderate either the relationship between stress and anxiety or the relationship between stress and GI symptoms. Additionally, vigorous activity did not moderate the relationship between stress and anxiety. However, vigorous activity was a significant moderator of the relationship between stress and GI symptoms, *Δ* $R^{2}$ = 0.037, *p* = 0.003. Conditional effects were examined at ± 1 SD. The association of perceived stress with GI symptoms was stronger at higher levels of vigorous activity, *b* = 1.44, *p* < 0.001, compared to lower levels of vigorous activity, *b* = 0.72, *p* < 0.001. Procedures specified by Bodner (2017) were used to make standardized comparisons of the conditional effects. The effects at each level of vigorous physical activity can be interpreted similarly to a partial correlation. At lower levels of vigorous physical activity, the standardized association between perceived stress and GI symptoms was 0.28; at higher levels of vigorous physical activity, the standardized association was 0.57.

### Mediating effects of gastrointestinal symptoms

**Figure 1:**
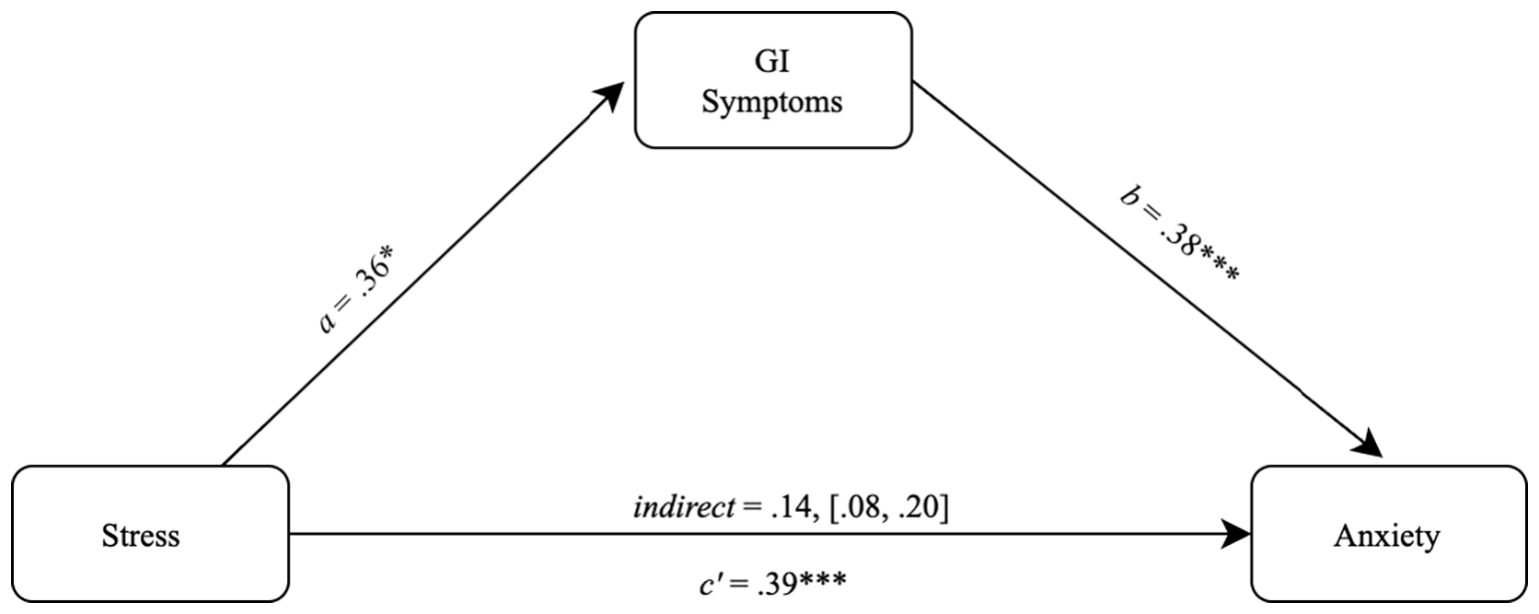
Mediation model of stress and anxiety through gastrointestinal symptoms. Effects are standardized; c’ represents direct effect; *p < 0.05, **p < 0.01, ***p < 0.001.

GI symptoms did not significantly mediate the relationship between diet and anxiety or the relationship between vigorous activity and anxiety. However, GI symptoms indirectly linked perceived stress and anxiety. The standardized indirect effect through GI symptoms was *b* = 0.14 [0.08, 0.20]. The standardized direct effect of stress with anxiety was *b* = 0.39, *p* < 0.001. The standardized pathway coefficients are depicted in Figure 1.

**Figure 2:**
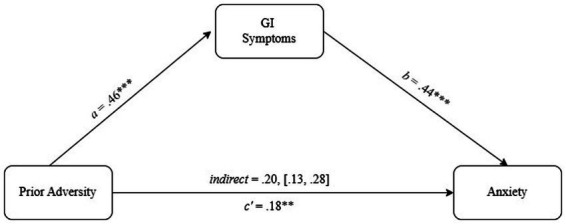
Mediation model of prior adversity and anxiety through gastrointestinal symptoms. Effects are standardized; c’ represents direct effect; *p < 0.05, **p < 0.01, ***p < 0.001.

GI symptoms also mediated the relationship between prior adversity and anxiety. The standardized indirect effect through GI symptoms was *b* = 0.20 [0.12, 0.28]. The standardized direct effect of prior adversity with anxiety was *b* = 0.18, *p* = 0.007. The standardized pathway coefficients are depicted in Figure 2.

### Stress and GI as serial mediators

**Figure 3:**
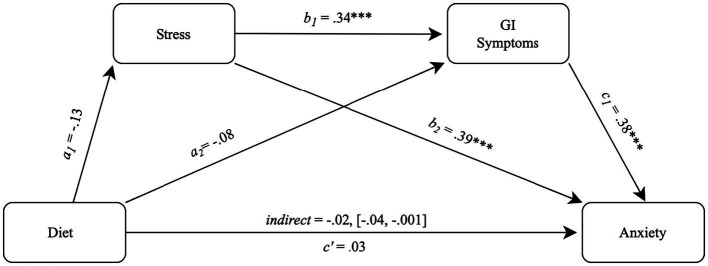
Serial mediation for diet to anxiety through stress and GI symptoms. Effects are standardized; c represents indirect effect; c’ represents direct effect; *p < 0.05, **p < 0.01, ***p < 0.001.

Stress and GI symptoms did not serially mediate the relationship between vigorous activity and anxiety. However, the indirect pathway through GI symptoms alone was significant, *b* = 0.06, [0.01, 0.14]. Stress and GI symptoms acted as serial mediators of the relationship between diet quality and anxiety. The standardized indirect effect of the serial pathway from diet quality to stress to GI symptoms to anxiety was *b* = −0.017, [−0.039, −0.0002]. The indirect pathway through stress alone was also significant, *b* = −0.05, [−0.11, −0.001]. The standardized pathway coefficients are depicted in Figure 3.

**Figure 4:**
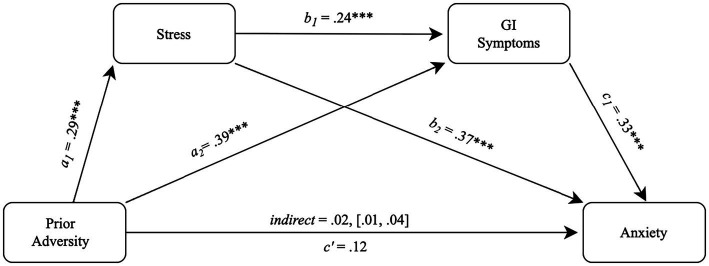
Serial mediation for prior adversity to anxiety through stress and GI symptoms. Effects are standardized; c represents indirect effect; c’ represents direct effect; *p < 0.05, **p < 0.01, ***p < 0.001.

Perceived stress and GI symptoms also served as serial mediators of the relationship between prior adversity and anxiety. The standardized indirect effect of the serial pathway from prior adversity to stress to GI symptoms to anxiety was *b* = 0.02, [0.01, 0.05]. There were also indirect effects of perceived stress as the sole mediator, *b* = 0.11, [0.06, 0.16], and GI symptoms as the sole mediator, *b* = 0.13, [0.06, 0.20]. The standardized pathway coefficients are depicted in Figure 4.

## Discussion

The present study aimed to investigate the gut-brain axis and multiple environmental stressors, including diet quality, physical activity levels, and prior adversities. Specifically, we focused on ways in which diet and physical activity, being external physical stressors, may moderate the relationship between perceived stress and anxiety, as well as the relationship between perceived stress and GI symptoms. We also aimed to research GI symptoms as a mediator between various stressors and anxiety. We further investigated GI symptoms in a serial mediation model with perceived stress and GI symptoms as links between external stressors and anxiety.

### Diet and gut-brain axis

While diet has shown relations with the gut-brain axis in previous literature (Madison and Kiecolt-Glaser, 2019; Malesza et al., 2021; Tomasello et al., 2016), little was found in our analyses. Diet was not shown to be related to anxiety and was not directly related to stress or GI symptoms. Additionally, diet did not moderate the relationships between stress and anxiety or stress and GI symptoms, which did not support our initial hypotheses. There was also no mediation found between diet and anxiety through GI symptoms, which contrasts previous literature that suggested probiotics in the diet are associated with better mental health outcomes (Hilimire et al., 2015; Ma et al., 2021; Nobile and Puoci, 2023; Tillisch et al., 2013). However, the literature is mixed when it comes to certain diet supplementations such as probiotics. For example, Slykerman et al. (2022) found that supplementation of a probiotic was not associated with mental health outcomes.

However, our hypothesis of the serial mediation from diet quality to stress to GI symptoms to anxiety was supported. This finding is in support of previous literature that has found low diet quality to be related to poor gut microbiomes, stress levels, and systemic inflammation (Madison and Kiecolt-Glaser, 2019; Malesza et al., 2021; Tomasello et al., 2016). As measured in the diet quality questionnaire, good quality diet towards promoting a healthy gut includes high fiber intake and low consumption of saturated fats in balance with unsaturated fats (Bailey and Holscher, 2018). Given that stress and GI symptoms have been shown to be related to anxiety (Hilimire et al., 2015; Mihić et al., 2023; Zou et al., 2023), we expected that diet and stress would be associated with gut-related issues, which in turn would be associated with anxiety scores.

Our results support the predicted relationships between stress, GI symptoms, and anxiety, but direct relations with diet were not found. The overall serial mediation effect was significant but small, which could have been due to the nature of our diet quality results. This may reflect limited variability within the sample, given a college student population, or the ability of the SFFFQ to detect necessary variations in diet. For example, 86.6% of our participants fell below the threshold of a good quality diet, which may have affected the power of our results. However, with the serial mediation being supported, potential implications of this research may be to promote healthier eating habits and education surrounding nutrition at college campuses to potentially improve mental health outcomes. Additionally, the small effect may suggest that clinicians should consider diet as a potential factor when evaluating patients for anxiety-related mental health issues. However, research with a more generalizable sample and alternative dietary measures could further elucidate these findings.

### Vigorous activity and gut-brain axis

Based on prior literature (Clauss et al., 2021; Gottschall et al., 2020; Hill et al., 2008; Vanbruggen et al., 2011), we predicted that high vigorous activity would strengthen the associations of stress with anxiety and stress with GI symptoms. Our results found that vigorous activity moderated the relationship between stress and GI symptoms but not the relationship between stress and anxiety. The finding of vigorous activity’s moderation of the relationship between stress and GI symptoms supports the research findings of Clauss et al. (2021), which found level of exercise to be associated with higher gut permeability. Higher gut permeability may lead to increased pathogens in the gut that may cause the inflammation associated with GI issues. These results also support the findings of Wilson (2018), which found perceived exertion in runners to be correlated with GI distress, along with anxiety being correlated with this GI distress. While stress is associated with GI issues through the processes of the autonomic nervous system (De Punder and Pruimboom, 2015), our results suggest that the addition of high vigorous activity strengthens this relationship.

Our research focused on the variable of perceived stress rather than physiological stress, which is often the stress variable associated with high-intensity activity. As discussed, physiological stress is related to the secretion of cortisol, which is why high cortisol levels are observed in response to vigorous activity (Gottschall et al., 2020; Hill et al., 2008; Vanbruggen et al., 2011). While the current study does not measure the physical outcomes of physiological stress, we expected to see similar results reflected in perceived stress when participants had higher vigorous activity. Our prediction was not supported, suggesting that perceived stress is a more chronic, long-term measure of stress as compared to physiological stress. This may not have been captured, given that our study was not longitudinal, and vigorous activity was only collected via self-report over a span of 1 week. Future research may want to examine perceived stress and physiological stress separately in the context of physical activity and anxiety. It is also important to note that high vigorous activity was not significantly correlated with anxiety, also indicating that the measure of perceived stress may not be related to vigorous levels of physical activity.

Relatedly, we aimed to explore whether adding GI symptoms would mediate the relationship between vigorous activity and anxiety, given relations found between vigorous activity and GI symptoms (Clauss et al., 2021) and GI issues with anxiety (Hilimire et al., 2015). The hypothesis that GI symptoms would mediate this relationship was not supported. Furthermore, serial mediation from vigorous activity to anxiety through stress and GI issues was also not significant.

### Prior adversity and gut-brain axis

As predicted, the relationship between prior adversity and anxiety was mediated by GI symptomology, in that higher levels of prior adversity were associated with higher levels of GI symptomology, which were associated with higher levels of anxiety. These findings support previous literature that has related prior adversity to anxiety (Kim et al., 2023; Goodman et al., 2022), as well as relations found between prior adversity and the autonomic nervous system (Kolacz et al., 2020). This relationship was further supported in a serial mediation from prior adversity to anxiety through stress and GI symptoms, in that greater prior adversity was associated with greater perceived stress, which was associated with greater GI symptoms, which in turn was related to increased anxiety. This further supports research relating prior adversities to stress and anxiety (Goodman et al., 2022; Kim et al., 2023), stress to GI symptoms (De Punder and Pruimboom, 2015), and GI symptoms to anxiety (Zou et al., 2023).

The findings of the serial mediation allow for a further understanding of potential directional relationships between these variables of the gut-brain axis in relation to prior adversity. The utilized scale captured prior adversity from childhood to adulthood, which may have encompassed both chronic and acute stressors, depending on the stressor and when it occurred. Our results suggest that experiencing prior adverse life events may be related to mental health through autonomic nervous system functioning. Relating prior adversity to the gut-brain axis allows for potential clinical implications for anxiety that is seemingly related to gut-health issues. Treating impacts of prior adversity through treatments related to trauma, such as mindfulness practices, cognitive behavioral therapy, or dialectical behavioral therapy, may be helpful in lessening anxiety and gut issues. More research is needed in this area to better understand ways in which treatments of prior adversity may impact these outcomes.

### Limitations

While the study results are potentially influential, it is important to point out the limitations. Notably, the final sample (*N* = 209) fell below the targeted sample size indicated by our power analyses surrounding moderation effects (*N* = 256) and small-effect mediation (*N* = 310). Failing to meet the target sample sizes reduced the study’s ability to detect small mediation and moderation effects. With that, our non-significant findings could be subject to type II error, particularly our findings regarding diet. Future studies should recruit larger samples to clarify associations between diet, stress, GI symptoms, and anxiety. We did, however, reach beyond our target sample size for detecting simple mediation with associations of *r* = 0.3 (*N* = 150), suggesting sufficient power for detecting effects of this magnitude.

Additionally, the sample was majority white, female, and young (Table 1). Most of the participants in this study were recruited from undergraduates at a southeastern university in the United States, which impacts the generalizability of our study. The processes of the gut-brain axis are multifaceted, suggesting that gender, racial, and age differences could impact results. Specifically, previous literature has found differences in gut microbiome composition depending on biological sex (Kim et al., 2020), race (Mallott et al., 2023), and age (Badal et al., 2020). It is important to acknowledge that individual differences could have played a role in gut composition and access to different types of food and physical activity resources.

Additionally, recruiting mainly from a college population may have had an impact on the diet quality scores overall. As mentioned above, 86.6% of the participants in our study fell below the threshold of a good quality diet. Our largely null results related to diet could be due to this lack of variability in the diet quality measure. College students may not be well educated on nutrition or may not be financially able to access healthy, nutrient-dense meals. Outside of the perspective of college students, we may also have seen low diet quality scores due to the study taking place in the United States where western diets are more common. As discussed in the background literature, the typical Western diet is harmful towards the gut microbiome due to the consumption of inflammatory foods and low fiber intake (Malesza et al., 2021). It may also be that the effects of diet would be more apparent in older populations as the chronic effects of diet may impact health outcomes over time (Chen et al., 2019; Govindaraju et al., 2018; Mao et al., 2024).

It is also important to acknowledge that our study was based on self-report survey data and is purely correlational. While directional analyses were used, the study cannot claim causation of the direction of these predictions. These correlations are suggestions as to the directional relationships of these variables.

### Future directions

The study has provided further insight into the relationships of physiological and environmental stressors with the gut-brain axis. However, the observed relationships need to be studied longitudinally to better understand the directional influence over time. This is especially true when examining dietary implications and chronic physical activity level implications. Future studies should focus on aspects of stressors, like components of diet and physical activity, separately and experimentally, to better understand relations with the gut-brain axis. This may be in the form of feeding studies or exercise studies in which participants are put on a schedule to record eating and physical activity habits.

With diet specifically, there are many components that could relate to one’s gut-brain axis through inflammation. Inflammation may not look the same for every participant given food sensitivities, autoimmune disorders, or allergies. Likewise, it may be beneficial to examine differing food groups and their associations with the gut-brain axis, such as a diet high in pre- and probiotic promoting foods that are fermented or rich in fiber. While the Mediterranean diet was emphasized in the background literature, it is also important to acknowledge that there may be other combinations of diet that are beneficial in promoting healthy gut microbiomes.

Concerning physical activity, future studies should focus further on the component of vigorous activity, as our study suggests it may be associated with GI symptoms. Specifically, it would be beneficial to research the gut-brain axis in endurance athletes as they are likely to be under the impact of chronic strenuous activity. Conversely, research is needed on sedentary lifestyles and their relation to the gut-brain axis and mental health outcomes.

## Conclusion

The previous literature and the unique findings of the current study suggest that factors such as diet and physical activity should be considered when examining gut-brain relations specific to stress, GI symptoms, and anxiety. With further investigation of these relationships, future clinicians may better understand how mental health interacts with factors of the autonomic nervous system through external stressors related to lifestyle (e.g., diet and physical activity) and adverse experiences. Future research should further investigate the directionality of these relationships more clearly through longitudinal study designs, especially given the bidirectional nature of the gut-brain axis.

## Data Availability

The datasets presented in this study can be found in online repositories. The names of the repository/repositories and accession number(s) can be found in the article/supplementary material.
